# Incidence and risk of venous thromboembolism according to primary treatment in women with ovarian cancer: A retrospective cohort study

**DOI:** 10.1371/journal.pone.0250723

**Published:** 2021-04-28

**Authors:** Jin-Sung Yuk, Banghyun Lee, Kidong Kim, Myoung Hwan Kim, Yong-Soo Seo, Sung Ook Hwang, Sang-Hee Yoon, Yong Beom Kim

**Affiliations:** 1 Department of Obstetrics and Gynecology, Sanggye Paik Hospital, School of Medicine, Inje University, Seoul, Republic of Korea; 2 Department of Obstetrics and Gynecology, Inha University Hospital, Inha University School of Medicine, Incheon, Republic of Korea; 3 Department of Obstetrics and Gynecology, Seoul National University Bundang Hospital, Seongnam-Si, Gyeonggi-Do, Republic of Korea; Chang Gung Memorial Hospital and Chang Gung University, Taoyuan, Taiwan, TAIWAN

## Abstract

**Objective:**

This study aimed to investigate incidence and risk for venous thromboembolism (VTE) according to primary treatment in women with ovarian cancer.

**Methods:**

We selected 26,863 women newly diagnosed with ovarian cancer between 2009 and 2018 from the Korean Health Insurance Review and Assessment Service databases. During the total follow-up period and the first six months after initiation of primary treatments, incidence and risk of VTE were evaluated according to primary treatment as no treatment, surgery, radiotherapy, or chemotherapy.

**Results:**

The mean follow-up period was 1285.5±6 days. The VTE incidence was highest in women who underwent chemotherapy (306 per 10,000 women). Among women who underwent surgery, VTE was highest in surgery with neoadjuvant chemotherapy (536 per 10,000 women), followed by surgery with adjuvant chemotherapy (360 per 10,000 women) and surgery alone (132 per 10,000 women). During the first 12 months, monthly incidence of VTE decreased. Compared with women with no treatment, risk of VTE significantly increased in women undergoing chemotherapy (HR 1.297; 95% CI, 1.08–1.557; P = 0.005) during the total follow-up period and decreased in women undergoing surgery (HR 0.557; 95% CI, 0.401–0.775; P<0.001) and radiotherapy (HR 0.289; 95% CI, 0.119–0.701; P = 0.006) during the first six months. Among women who underwent surgery, VTE risk significantly increased in surgery with neoadjuvant chemotherapy (HR 4.848; 95% CI, 1.86–12.632; P = 0.001) followed by surgery with adjuvant chemotherapy (HR 2.807; 95% CI, 1.757–4.485; P<0.001) compared with surgery alone during the total follow-up period and in surgery with neoadjuvant chemotherapy (HR 4.223; 95% CI, 1.37–13.022; P = 0.012) during the first six months.

**Conclusions:**

In this large Korean cohort study, incidence and risk of VTE were highest in women with ovarian cancer who underwent chemotherapy and surgery with neoadjuvant chemotherapy as a primary cancer treatment. Incidence of VTE decreased over time.

## Introduction

Venous thromboembolism (VTE), comprising deep vein thrombosis (DVT) and pulmonary embolism (PE), is a common complication of gynecologic cancer and is associated with high morbidity and mortality [1]. Incidence of VTE in women with gynecologic cancer has been reported to be 0%-41.5% because of heterogenous study designs and population [1–23]. Although current guidelines guide prophylaxis and treatment of VTE in women with cancer, management of VTE in women with gynecologic cancers is mainly based on studies of solid cancers [1, 24–27].

Some studies have reported that incidence of VTE in women with gynecologic cancers varies by therapeutic modality [2–6, 13, 14, 18–21, 23, 28]. Incidence of VTE in gynecologic cancers ranged between 0% and 13.8% among women undergoing surgery, and was reported as 11% and 33.8% among women undergoing chemotherapy [3–6, 13, 14, 18–21, 23, 28]. Moreover, incidence of VTE in women undergoing gynecologic brachytherapy was 1.2% [2].

Epithelial ovarian cancer (EOC), which accounts for 90% of ovarian cancer, is the most common cause of gynecologic cancer death, inducing 14 000 deaths every year in the USA [29, 30]. Most EOC (75%) presents as advanced (stage III or IV) disease, and most women with advanced disease undergo repeated episodes of recurrent disease with progressively shorter disease-free interval [31]. Therefore, most women with EOC receive repeated chemotherapy after the standard of care (debulking surgery and platinum-based cytotoxic chemotherapy). Advanced stage of cancer and major surgery are associated with increased risk of VTE [25]. A large number of chemotherapy cycles might be also associated with increased risk of VTE.

Large-scale studies evaluating incidence and risk of VTE according to therapeutic modality in different types of gynecologic cancers may guide prophylaxis and treatment of VTE according to gynecologic cancer type. Moreover, using the nationwide population-based database for a cohort study can provide much more data compared with cohorts based on multiple institutions. Therefore, this study aimed to investigate the incidence and risk for VTE according to primary treatment in women with ovarian cancer using Korean Health Insurance Review & Assessment Service (HIRA) data.

## Materials and methods

### Study population and design

South Korea has a universal health coverage system, the National Health Insurance, which covers approximately 98% of the overall Korean population [32, 33]. National Health Insurance offers medical insurance services for most diseases, except cosmetic surgery, and includes medical information such as sex, age, region, insurance type, low-income household, diagnostic code, surgery code, and prescription code [32]. In particular, diagnosis codes for cancer have high accuracy because women with cancer receive additional medical payment discounts. The HIRA is a neutral entity that assesses almost all medical payments between the Korean National Health Insurance Service (NHIS) and medical institutions and shares most NHIS data [32]. The claims data of the HIRA represent 23 million women per year. This is a retrospective cohort study using insurance data from the HIRA between January 1, 2007 and December 31, 2018.

Diagnostic codes, surgery codes, and prescription codes to select eligible women were obtained from the 10^th^ revision of the International Statistical Classification of Diseases and Related Health Problems (ICD-10), the Health Insurance Medical Care Expenses (2017 and 2018 version), and the HIRA Drug Ingredients Codes. Women with ovarian cancer were defined as those who had five or more diagnostic codes for ovarian cancer (ICD-10: C56x) and did not have diagnostic codes for other cancers between 2007 and 2018 based on data from the Korea Central Cancer Registry as a reference. Among women diagnosed with ovarian cancer, those who had diagnostic codes for VTE (ICD-10: I80.2, I80.3, I26) before the first C56x codes were excluded. Women who had C56x codes between 2007 and 2008 were also excluded to select only women with newly diagnosed ovarian cancer (wash out period). Women with VTE were defined as those who received more than two prescriptions for anticoagulants simultaneous with diagnostic codes for VTE after initiation of primary cancer treatment. In the absence of primary cancer treatment, women with VTE were defined as those who received more than two prescriptions for anticoagulants simultaneous with diagnostic codes for VTE from the date of the first diagnostic code for ovarian cancer. Deep vein thrombosis (DVT) and pulmonary embolism (PE) were defined as women who received more than two prescriptions for anticoagulants simultaneous with diagnostic codes of I80.2 or I80.3 and I26, respectively.

Age was classified in intervals of 5 years. Low socioeconomic status (SES) was defined as women with Medicaid as the form of Korean National Health Insurance. The Charlson Comorbidity Index (CCI) was calculated based on Quan’s method using data between 365 days and 1 day before the first diagnostic date of ovarian cancer [34]. Primary treatments were defined as treatments performed first after diagnosis of ovarian cancer and included no treatment, surgery, radiotherapy, and chemotherapy. In women who underwent no treatment, incidence of VTE after diagnosis of ovarian cancer was evaluated. Surgery was defined by surgery codes for salpingo-oophorectomy (bilateral or unilateral) or ovarian cystectomy and/or total hysterectomy simultaneous with diagnostic codes of ovarian cancer. If two or more surgeries were performed, the first surgery was counted as the primary treatment. Neoadjuvant chemotherapy was defined as that performed right before primary surgery, and adjuvant chemotherapy was defined as that performed within 6 weeks after primary surgery. Radiotherapy was defined as prescription codes for radiotherapy [external beam radiation therapy (EBRT) or concurrent chemoradiation therapy (CCRT)] simultaneous with diagnostic codes for ovarian cancer. Chemotherapy was defined as prescription codes for chemotherapy (bevacizumab, carboplatin, cisplatin, cyclophosphamide, docetaxel, fluorouracil, gemcitabine, ifosfamide, irinotecan, liposomal doxorubicin, mitomycin, paclitaxel, topotecan) simultaneous with the diagnostic code for ovarian cancer. Pharmacologic thromboprophylaxis for VTE was defined as more than two prescriptions for anticoagulants without diagnostic codes for VTE after initiation of primary treatment or from the date of ovarian cancer diagnosis (if treatment was not performed). Anticoagulants were comprised of low-molecular weight heparin (LMWH), unfractionated heparin (UFH), warfarin, aspirin, direct oral anticoagulants (DOAC), and fondaparinux.

### Statistical analyses

Categorical variables were analyzed with the Chi-square test and Fisher’s exact test, while continuous variables were analyzed with Student’s t-test and Mann-Whitney U test. The Cox Proportional Hazard Regression model with or without adjustment for confounding factors was used to analyze associations between variables and VTE occurrence. All statistical analyses were conducted with two-tailed tests, and a *P* value <0.05 was considered to indicate statistical significance. When there was a missing value, the mean imputation method was used. All statistical analyses were performed using SAS® Enterprise Guide® version 6.1 (SAS Institute, Inc., Cary, NC, USA) and R version 3.0.2 (R Foundation for Statistical Computing, Vienna, Austria).

### Ethics

Based on South Korea’s Bioethics and Safety Act, because the HIRA dataset uses anonymous identification codes to protect personal information, approval of this study was waived by the Institutional Review Board of Inha University Hospital (No. 2019-11-007) on November 25, 2019, and informed consent was not required. The HIRA does not have responsibility for the results of this study.

## Results

**Fig 1:**
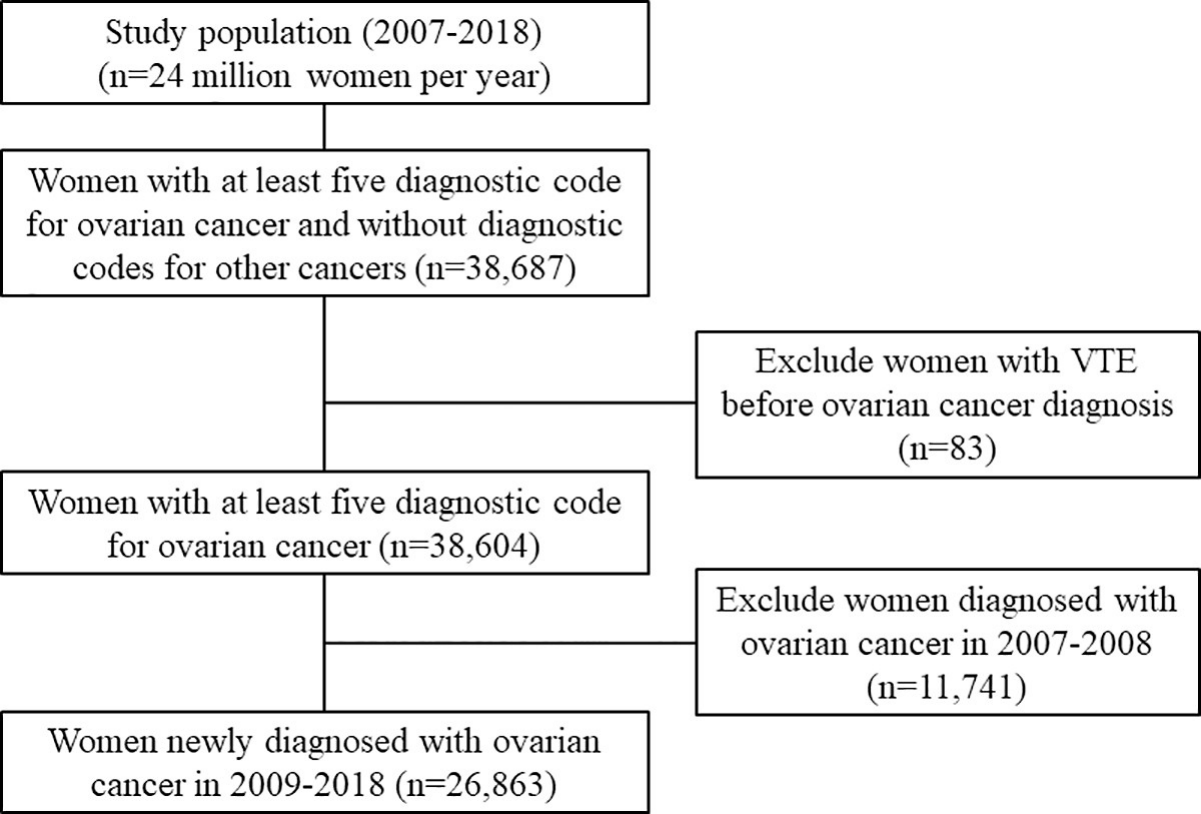
Flow chart showing selection of eligible women.

Data from 38,687 women who had five or more diagnostic codes for ovarian cancer between 2007 and 2018 were extracted. Of these, 26,863 women newly diagnosed with ovarian cancer since 2009 were selected (Fig 1).

### Characteristics of women with ovarian cancer

Characteristics of women with ovarian cancer are shown in detail in Table 1. VTE occurred in 2.6% and 1.7% of women during the total follow-up period and during the first six months after initiation of primary treatments, respectively. The mean follow-up period was 1285.5±6 days.

**Table 1:** Characteristics of women with ovarian cancer in HIRA claims data of 2009–2018.

	The total follow-up period				The first six months			
	VTE (-)	VTE (+)	Total		VTE (-)	VTE (+)	Total	
	n = 26,161 (97.4%)	n = 702 (2.6%)	n = 26,863 (100%)	*P* value	n = 26,418 (98.3%)	n = 445 (1.7%)	n = 26,863 (100%)	*P* value
**Mean age (years)**	52.8±0.1	58.7±0.4	53.0±0.1	<0.001 ^a^	52.9±0.1	59.1±0.6	53.0±0.1	<0.001 ^a^
**SES**				0.069				0.019
** Mid- or high-SES**	24,962 (95.4)	680 (96.9)	25,642 (95.5)		25,207 (95.4)	435 (97.8)	25,642 (95.5)	
** Low SES**	1,199 (4.6)	22 (3.1)	1,221 (4.5)		1,211 (4.6)	10 (2.2)	1,221 (4.5)	
**CCI**				<0.001				<0.001
** 0**	12,086 (46.2)	238 (33.9)	12,324 (45.9)		12,164 (46)	160 (36)	12,324 (45.9)	
** 1**	2,764 (10.6)	77 (11)	2,841 (10.6)		2,799 (10.6)	42 (9.4)	2,841 (10.6)	
** 2**	3,995 (15.3)	117 (16.7)	4,112 (15.3)		4,043 (15.3)	69 (15.5)	4,112 (15.3)	
** 3**	1,195 (4.6)	33 (4.7)	1,228 (4.6)		1,207 (4.6)	21 (4.7)	1,228 (4.6)	
** Over 4**	6,121 (23.4)	237 (33.8)	6,358 (23.7)		6,205 (23.5)	153 (34.4)	6,358 (23.7)	
**Year of cancer diagnosis**				<0.001				<0.001
** 2009**	2,158 (8.2)	40 (5.7)	2,198 (8.2)		2,176 (8.2)	22 (4.9)	2,198 (8.2)	
** 2010**	2,235 (8.5)	52 (7.4)	2,287 (8.5)		2,260 (8.6)	27 (6.1)	2,287 (8.5)	
** 2011**	2,343 (9)	55 (7.8)	2,398 (8.9)		2,365 (9)	33 (7.4)	2,398 (8.9)	
** 2012**	2,431 (9.3)	66 (9.4)	2,497 (9.3)		2,465 (9.3)	32 (7.2)	2,497 (9.3)	
** 2013**	2,559 (9.8)	68 (9.7)	2,627 (9.8)		2,584 (9.8)	43 (9.7)	2,627 (9.8)	
** 2014**	2,785 (10.6)	61 (8.7)	2,846 (10.6)		2,812 (10.6)	34 (7.6)	2,846 (10.6)	
** 2015**	2,796 (10.7)	99 (14.1)	2,895 (10.8)		2,835 (10.7)	60 (13.5)	2,895 (10.8)	
** 2016**	3,061 (11.7)	116 (16.5)	3,177 (11.8)		3,099 (11.7)	78 (30)	3,177 (11.8)	
** 2017**	3,048 (11.7)	92 (13.1)	3,140 (11.7)		3,074 (11.6)	66 (14.8)	3,140 (11.7)	
** 2018**	2,745 (10.5)	53 (7.5)	2,798 (10.4)		2,748 (10.4)	50 (11.2)	2,798 (10.4)	
**Primary treatment** ^**b**^				0.003				<0.001
** No treatement** ^**c**^	12,451 (47.6)	321 (45.7)	12,772 (47.5)		12,534 (47.4)	238 (53.5)	12,772 (47.5)	
** Surgery ± adjuvant chemotherapy**	4,041 (15.4)	94 (13.4)	4,135 (15.4)		4,093 (15.5)	42 (9.4)	4,135 (15.4)	
** Radiotherapy ± adjuvant chemotherapy**	1,220 (4.7)	20 (2.8)	1,240 (4.6)		1,235 (4.7)	5 (1.1)	1,240 (4.6)	
** Chemotherapy**	8,449 (32.3)	267 (38)	8,716 (32.4)		8,556 (32.4)	160 (36)	8,716 (32.4)	
**Surgery ± adjuvant chemotherapy**								
** Methods of surgery**								
** Total hysterectomy ± BSO or USO or ovarian cystectomy**	2,573 (63.7)	77 (81.9)	2,650 (64.1)	<0.001	2,616 (63.9)	34 (81)	2,650 (64.1)	0.022
** BSO or USO or ovarian cystectomy**	1,468 (36.3)	17 (18.1)	1,485 (35.9)	<0.001	1,477 (36.1)	8 (19)	1,485 (35.9)	0.022
** Other surgeries** ^**d**^	1,226 (8.6)	42 (4.3)	1,268 (8.5)	0.003	1,255 (8.6)	13 (2.4)	1,268 (8.5)	1 ^e^
** Types of surgical treatment**				<0.001				<0.001
** Surgery alone**	2,462 (60.9)	33 (35.1)	2,495 (60.3)		2,478 (60.5)	17 (40.5)	2,495 (60.3)	
** Surgery + adjuvant chemotherapy**	1,473 (36.5)	55 (58.5)	1,528 (37)		1,508 (36.8)	20 (47.6)	1,528 (37)	
** Neoadjuvant chemotherapy + surgery ± adjuvant chemotherapy**	106 (2.6)	6 (6.4)	112 (2.7)		107 (2.6)	5 (11.9)	112 (2.7)	
**Chemotherapy**								
** Platinum**	7,313 (86.6)	240 (89.9)	7,553 (86.7)	0.115	7,404 (86.5)	149 (93.1)	7,553 (86.7)	0.015
** Other agents**	1,000 (11.8)	22 (8.2)	1,022 (11.7)	0.072	1,012 (11.8)	10 (6.3)	1,022 (11.7)	0.03
** Bevacizumab**	1,522 (18)	85 (31.8)	1,607 (18.4)	<0.001	1,564 (18.3)	43 (26.9)	1,607 (18.4)	0.006
**Time between primary treatments and VTE diagnosis (days)**		302.2±21.6				67.6±3.7		

BSO, bilateral salpingo-oophorectomy; CCI, Charlson comorbidity index; HIRA, health insurance review & assessment Service; n, number; SES, socioeconomic status; USO, unilateral salpingo-oophorectomy; VTE, venous thromboembolism.

All values are expressed as mean ± standard error or number (%).

^a^ The Mann-Whitney U test was used for this analysis.

^b^ Primary treatments refers to the first cancer treatments.

^c^ Incidence of VTE after diagnosis of ovarian cancer was evaluated.

^d^ Appendectomy, bowel resection, cholecystectomy, end-to-end ureteroureterostomy, pancreatectomy, partial gastrectomy, partial hepatectomy, pelvic and/or para-aortic lymph node dissection, splenectomy, stripping of other peritoneal surfaces, stripping of the diaphragm, ureteroneocystostomy.

^e^ The Fisher’s exact test was used for this analysis.

### Incidence of VTE in various primary treatments in women with ovarian cancer

VTE occurred in 261 per 10,000 women during the total follow-up period and in increasing order of frequency with chemotherapy, no treatment, surgery, and radiotherapy. VTE occurred in 166 per 10,000 women during the first six months, a similar frequency to chemotherapy and no treatment followed by surgery and then radiotherapy. Regardless of the period after initiation of primary treatments, VTE, DVT, and PE, thromboembolism occurred in increasing order of frequency with neoadjuvant chemotherapy/surgery with or without adjuvant chemotherapy, surgery with adjuvant chemotherapy, and surgery alone (Table 2).

**Table 2:** Incidence (per 10,000 women) of VTE in various primary treatments in women with ovarian cancer (HIRA claims data of 2009–2018).

	The total follow-up period						The first six months						Total cases
	VTE		DVT		PE		VTE		DVT		PE		
	Count	Incidence	Count	Incidence	Count	Incidence	Count	Incidence	Count	Incidence	Count	Incidence	
**Primary treatment** ^**a**^													
** No treatment** ^**b**^	321	251	147	115	193	151	238	186	106	83	146	114	12,772
** Surgery**	94	227	53	128	49	119	42	102	17	41	28	68	4,135
** Surgery alone**	33	132	21	84	14	56	17	68	8	32	9	36	2,495
** Surgery + adjuvant chemotherapy**	55	360	29	190	31	203	20	131	7	46	15	98	1,528
** Neoadjuvant chemotherapy + surgery ± adjuvant chemotherapy**	6	536	3	268	4	357	5	446	2	179	4	357	112
** Radiotherapy**	20	161	10	81	11	89	5	40	3	24	3	24	1,240
** Chemotherapy**	267	306	99	114	177	203	160	184	57	65	107	123	8,716
**Total cases**	702	261	309	115	430	160	445	166	183	68	284	106	26,863

DVT, deep vein thrombosis; HIRA, Health Insurance Review & Assessment Service; PE, pulmonary embolism; VTE, venous thromboembolism.

^a^ Primary treatments refers to the first cancer treatments.

^b^ Incidence of VTE after diagnosis of ovarian cancer was evaluated.

**Fig 2:**
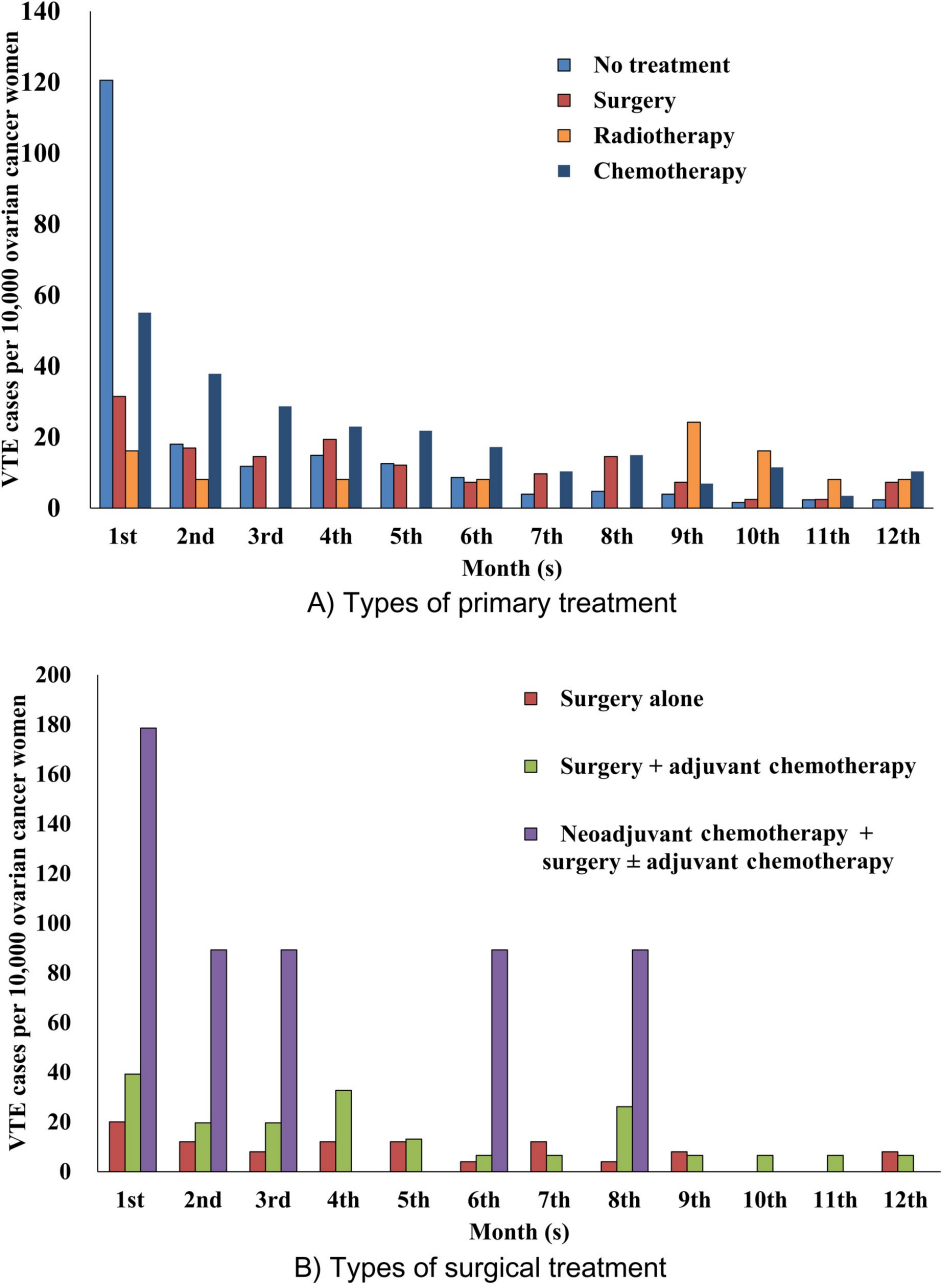
Incidence of VTE for the first 12 months after initiation of primary treatment in women with ovarian cancer (HIRA claims data of 2009–2018). In women who underwent no treatment, incidence of VTE after diagnosis of ovarian cancer was evaluated.

During the first 12 months after initiation of primary treatment, monthly incidence of VTE was highest with chemotherapy and decreased over time with no treatment, surgery, and chemotherapy other than radiotherapy with the highest frequency in the ninth month. Moreover, monthly incidence of VTE occurred in increasing order of frequency with neoadjuvant chemotherapy/surgery with or without adjuvant chemotherapy, surgery with adjuvant chemotherapy, and surgery alone, and decreased over time (Fig 2A and 2B).

During the total follow-up period, the highest incidence occurred between 50 and 70 years for no treatment and chemotherapy, between 50 and 55 years for surgery, and did not exist for radiotherapy (S1 Fig).

### Association of risk factors with VTE occurrence in women with ovarian cancer

During the total follow-up period, when adjusted for other confounding factors including types of primary treatment, risk of VTE significantly increased according to increased age per 5 years, CCI, and year of ovarian cancer diagnosis. Risk of VTE significantly increased with use of pharmacologic thromboprophylaxis and decreased in low SES compared with mid- or high-SES. Moreover, only chemotherapy was associated with a significant increase in VTE risk, especially for PE compared with no treatment (VTE: HR 1.297; 95% CI, 1.08–1.557; *P* = 0.005) (PE: HR 1.283; 95% CI, 1.02–1.615: *P* = 0.033). Risks of VTE related to various factors were similar during the first six months after initiation of primary treatment and over the total follow-up period. However, during the first six months, pharmacologic thromboprophylaxis was not a risk factor for VTE, and surgery and radiotherapy were associated with significant decrease in VTE risk compared with no treatment (surgery: HR 0.557; 95% CI, 0.401–0.775; *P*<0.001) (radiotherapy: HR 0.289; 95% CI, 0.119–0.701; *P* = 0.006) (Table 3).

**Table 3:** Association of risk factors with VTE occurrence in women with ovarian cancer (HIRA claims data of 2009–2018).

	The total follow-up period		The first six months	
	VTE		VTE	
	HR (95% CI)	*P* value	HR (95% CI)	*P* value
**Unadjusted HR**				
** Age per 5 years**	1.209 (1.175–1.243)	<0.001	1.2 (1.158–1.243)	<0.001
** Low SES** ^**a**^	0.737 (0.482–1.126)	0.158	0.505 (0.27–0.945)	0.033
** CCI**	1.104 (1.079–1.129)	<0.001	1.094 (1.063–1.125)	<0.001
** Year of cancer diagnosis**	1.129 (1.095–1.163)	<0.001	1.131 (1.091–1.173)	<0.001
** Pharmacologic thromboprophylaxis**	1.72 (1.483–1.996)	<0.001	1.207 (1.002–1.455)	0.048
** Types of primary treatment** ^**b**^^,^^**c**^				
** Surgery**	0.882 (0.701–1.11)	0.283	0.531 (0.383–0.737)	<0.001
** Radiotherapy**	1.044 (0.664–1.642)	0.852	0.287 (0.118–0.695)	0.006
** Chemotherapy**	1.768 (1.495–2.09)	<0.001	1.168 (0.953–1.43)	0.134
** Types of surgical treatment** ^**d**^				
** Surgery + adjuvant chemotherapy**	3.526 (2.271–5.473)	<0.001	1.936 (1.014–3.696)	0.045
** Neoadjuvant chemotherapy + surgery ± adjuvant chemotherapy**	8.346 (3.432–20.297)	<0.001	7.297 (2.691–19.782)	<0.001
**Adjusted HR1** ^**e**^				
** Age per 5 years**	1.177 (1.141–1.214)	<0.001	1.185 (1.14–1.231)	<0.001
** Low SES**	0.577 (0.377–0.885)	0.012	0.386 (0.206–0.724)	0.003
** CCI**	1.058 (1.033–1.084)	<0.001	1.062 (1.031–1.095)	<0.001
** Year of cancer diagnosis**	1.097 (1.062–1.134)	<0.001	1.126 (1.082–1.171)	<0.001
** Pharmacologic thromboprophylaxis**	1.175 (1.003–1.377)	0.046	0.858 (0.703–1.048)	0.133
** Types of primary treatment** ^**b**^^,^^**c**^				
** Surgery**	0.906 (0.719–1.142)	0.405	0.557 (0.401–0.775)	<0.001
** Radiotherapy**	0.984 (0.624–1.551)	0.945	0.289 (0.119–0.701)	0.006
** Chemotherapy**	1.297 (1.08–1.557)	0.005	0.844 (0.676–1.053)	0.133
**Adjusted HR2** ^**f**^				
** Age per 5 years**	1.222 (1.119–1.334)	<0.001	1.238 (1.089–1.408)	0.001
** Low SES**	0.23 (0.032–1.657)	0.145	0 (0-Infinite)	0.996
** CCI**	1.023 (0.952–1.1)	0.529	0.997 (0.89–1.117)	0.963
** Year of cancer diagnosis**	1.071 (0.977–1.173)	0.142	1.131 (0.991–1.291)	0.068
** Pharmacologic thromboprophylaxis**	1.119 (0.726–1.724)	0.611	0.75 (0.392–1.436)	0.385
** Types of surgical treatment** ^**d**^				
** Surgery + adjuvant chemotherapy**	2.807 (1.757–4.485)	<0.001	1.512 (0.749–3.052)	0.249
** Neoadjuvant chemotherapy + surgery ± adjuvant chemotherapy**	4.848 (1.86–12.632)	0.001	4.223 (1.37–13.022)	0.012

CCI, Charlson comorbidity index; CI, confidence interval; DVT, deep vein thrombosis; HIRA, health insurance review & assessment service; HR, hazard ratio; SES, socioeconomic status; PE: pulmonary embolism; VTE, venous thromboembolism.

^a^ The reference was mid- or high-SES.

^b^ Primary treatment refers to the first cancer treatment.

^c^ The reference was no treatment. In women who underwent no treatment, incidence of VTE after diagnosis of ovarian cancer was evaluated.

^d^ The reference was surgery alone.

^e^ The data were adjusted for all risk factors (age per 5 years, low SES, CCI, year of ovarian cancer diagnosis, pharmacologic thromboprophylaxis, and types of primary treatment).

^f^ The data were adjusted for all risk factors (age per 5 years, low SES, CCI, year of ovarian cancer diagnosis, pharmacologic thromboprophylaxis, and types of surgical treatment).

During the total follow-up period and the first six months, when women receiving surgery were analyzed and adjusted for other confounding factors including types of surgical treatment, risk of VTE significantly increased according to increased age per 5 years. During the total follow-up period, all types of surgical treatment were associated with significant increase in VTE risk, especially for DVT and PE compared with surgery alone [surgery with adjuvant chemotherapy: (VTE: HR 2.807; 95% CI, 1.757–4.485; *P*<0.001) (DVT: HR 3.179; 95% CI, 1.602–6.309: *P*<0.001) (PE: HR 2.129; 95% CI, 0.871–5.205: *P* = 0.098)] [neoadjuvant chemotherapy/surgery with or without adjuvant chemotherapy: (VTE: HR 4.848; 95% CI, 1.86–12.632; P = 0.001) (DVT: HR 5.436; 95% CI, 1.589–18.599: *P* = 0.007) (PE: HR 6.364; 95% CI, 1.666–24.306: P = 0.007)]. During the first six months, only neoadjuvant chemotherapy/surgery with or without adjuvant chemotherapy was associated with significant increase in VTE risk compared with surgery alone (HR 4.223; 95% CI, 1.37–13.022; *P* = 0.012) (Table 3).

### Incidence of VTE according to use of prophylactic anticoagulants after various primary treatments in women with ovarian cancer

During the total follow-up period, 47.3% women with VTE did not use prophylactic anticoagulants (45% of DVT and 49.8% of PE) and 52.7% did use prophylactic anticoagulants (55% of DVT and 50.2% of PE). Incidence of VTE was 0.2% (0.9% of DVT and 1.4% of PE) in women who did not use prophylactic anticoagulants and 3.3% (1.5% of DVT and 1.9% of PE) in women who did use prophylactic anticoagulants (S1 Table).

Prescribed prophylactic anticoagulants were aspirin (51.0%), UFH (49.2%), DOAC (8.2%), LMWH (6.9%), and warfarin (6.6%). DOAC was the most frequently used therapeutic anticoagulant (59.0%), and 5.3% of women with VTE used an inferior vena cava filter (S2 Table).

## Discussion

Only a few studies have reported an incidence of VTE in women with ovarian cancer, ranging from 1.2% - 39.3% [7, 16–18, 22]. In our study, incidence of VTE in women with ovarian cancer was lower than in previous studies, supporting a lower incidence of VTE in Asian patients than in the Western population (Table 1) [35]. Moreover, it was reported that the prevalence of women with body mass index (BMI) ≥25.0 kg/m^2^ was14% in Korean women aged between 19 and 79 years in 2016 and was 78% in American women aged between 20 and 74 years in 2015–2016 [36, 37]. These differences may partially explain the low incidence of VTE in Korean women.

In one ovarian cancer cohort (n = 13,031), person-time incidence rate of VTE decreased over 2 years after cancer diagnosis; in another ovarian cancer cohort (n = 1,989), the absolute rate of VTE decreased over a median 2 years [7, 17]. Other cohort studies have reported that incidence of VTE in women with gynecologic cancers decreased over time for 30 days (n = 175) and 90 days (n = 4,158) from cancer surgery [38, 39]. In one cancer cohort with claims database analysis (n = 17,284), the incidence of VTE decreased over time during the 12 months after initiation of chemotherapy [28]. Corresponding to these findings, our study demonstrated that incidence of VTE decreased over time for all types of primary cancer treatments and regardless of receiving neoadjuvant chemotherapy or adjuvant chemotherapy among women who underwent surgery based on monthly incidence of VTE during the first 12 months (Fig 2A and 2B).

A cohort study reported that absence of cancer treatment was a risk factor for VTE in women with ovarian cancer (n = 328) [16]. In our study, incidence of VTE was 2.5% during the total follow-up period and 1.9% during the first six months among women who did not undergo treatment. A large-scale claims data study reported a 1.2% incidence of VTE within 5 weeks after major surgery in women with ovarian cancer (n = 11,491) [18]. In two ovarian cancer studies, incidence of VTE was 3.3% in women undergoing neoadjuvant chemotherapy and interval debulking surgery (n = 270), and cumulative incidence of VTE was 7.5% for 30 days and 13.8% for 6 months in women undergoing primary debulking surgery (n = 860) [20, 21]. In our study, among women who underwent surgery for ovarian cancer, incidence of VTE was 2.3% during the total follow-up period and 1% during the first six months. This incidence increased in the order of surgery with neoadjuvant chemotherapy, surgery with adjuvant chemotherapy, and surgery alone, regardless of the time period after initiation of primary cancer treatments, VTE, DVT, and PE. Moreover, risk of VTE increased in women who underwent surgery with neoadjuvant chemotherapy or surgery with adjuvant chemotherapy compared to those with surgery alone, showing increase only in women who underwent surgery with neoadjuvant chemotherapy during the first six months. A prospective study reported that prior pelvic radiation therapy was a risk factor for postoperative DVT in women undergoing major gynecologic surgery (n = 411) [40]. In our study, incidence of VTE was 1.6% during the total follow-up period and 0.4% during the first six months among women who underwent radiotherapy (EBRT or CCRT). A cancer cohort study with claims database analysis reported an 11% incidence of VTE during the 12 months after initiation of chemotherapy in women with ovarian cancer (n = 1,880) [28]. A retrospective study reported that VTE in women with ovarian cancer undergoing cytoreductive surgery and adjuvant chemotherapy (n = 57) occurred in only women receiving bevacizumab with carboplatin and paclitaxel (8.8%) [19]. In our study, incidence of VTE was 3.1% during the total follow-up period and 1.8% during the first six months among women who underwent chemotherapy. Bevacizumab was more frequently used in women with VTE than in women without VTE showing more frequent use of platinum-based agents and bevacizumab in women with VTE during the first six months. In one ovarian cancer cohort (n = 13,031), risk of VTE decreased in women who underwent major surgery compared to women who did not [7]. In our study, incidence of VTE increased in the order of chemotherapy, no treatment, surgery, and radiotherapy regardless of the time period after initiation of primary cancer treatments, VTE, DVT and PE. Moreover, only chemotherapy was associated with increase in VTE risk compared with no treatment, showing decrease in surgery and radiotherapy during the first six months (Tables 1–3).

In one ovarian cancer cohort (n = 13,031), women older than 45 years had increased risk of VTE compared to women younger than 45 years (HR: 1.5–1.9) [7]. In another ovarian cancer cohort (n = 1,989), the absolute rate of VTE increased in women older than 60 years (20 per 1,000 person years vs 43 per 1,000 person years) [17]. In our study, regardless of time period after initiation of primary treatments, women with VTE were older than women without VTE, and risk of VTE increased with increasing age, showing the highest incidence mainly between 50 and 70 years in various primary treatments (Tables 1 and 3 and S1 Fig).

During the total follow-up period after initiation of primary treatments, 52.7% of women with VTE received prophylactic anticoagulants in our study. The incidence and risk of VTE were higher in women who used prophylactic anticoagulants compared to women who did not, showing no difference between the groups of women undergoing surgery. During the first six months, risk of VTE was similar in women with or without prophylactic anticoagulant treatments. These findings suggest that women exposed to known risk factors of VTE, such as old age, high CCI, chemotherapy, surgery with adjuvant chemotherapy, and surgery with neoadjuvant chemotherapy, might receive prophylactic anticoagulants more frequently. These women might be still at high risk of VTE with time although they used prophylactic anticoagulant treatments (Table 3 and S1 Table).

This study used a large-scale cohort to research the incidence and risk for VTE according to primary treatment in women diagnosed with ovarian cancer. This is the first study that investigated this topic in women with ovarian cancer. Limitations of this study relate to the use of claim data without review of medical records. The women included in this study were categorized based on diagnostic and prescription codes, without review of medical record. Hence, relevant women might not have been included or some unqualified women might not have been excluded due to erroneous codes. To minimize the error due to selection based on incorrect medical records, women without a prescription code or with only one prescription code for anticoagulants were not considered women with VTE. In the Korean national health insurance system, women diagnosed with VTE regardless of symptoms may be treated with anticoagulants; thus, there would be few women diagnosed with VTE and not treated. Furthermore, this study excluded women diagnosed with VTE prior to being diagnosed with ovarian cancer or administered primary cancer treatment. The order of diagnosis of VTE or ovarian cancer and initiation of primary cancer treatment can be reversed in real settings if they occur over a short time. However, our criteria were used to clarify relationships between VTE and ovarian cancer or primary cancer treatment. Finally, this study could not research the relationships between VTE and BMI, stage or histologic type of ovarian cancer, residual tumor, or number of cycles of chemotherapy because the HIRA dataset does not provide information about stage, histology, and surgical finding, uses anonymous identification codes and restricts access to the information. But this study, although not adjusted for cancer stage and histology, reveals that in women with ovarian cancer, primary treatment may present the risk of VTE compared with a no treatment group comprised of a large population with any stage and histology.

## Conclusions

Based on a large Korean cohort study, we evaluated the incidence and risk of VTE, and time course to VTE occurrence according to primary treatment in women with ovarian cancer. Incidence and risk of VTE were highest in women who underwent chemotherapy and surgery with neoadjuvant chemotherapy as a primary cancer treatment. Moreover, incidence of VTE decreased over time in each primary cancer treatment and surgical treatment. Our study also indicates that women at high risk for VTE might remain high risk despite pharmacologic thromboprophylaxis. The results of our study contribute to knowledge and management of VTE in women with ovarian cancer although the HIRA dataset has limitation in available information and access to the information, and uses anonymous identification codes for information. Prospective studies are needed to confirm these results.

## Supporting information

S1 Fig Incidence of VTE according to age in women with ovarian cancer (HIRA claims data of 2009–2018). (TIF)

S1 Table Incidence of VTE according to use of prophylactic anticoagulants in women with ovarian cancer (HIRA claims data of 2009–2018). (DOCX)

S2 Table Methods for prophylaxis and treatment of VTE in women with ovarian cancer (HIRA claims data of 2009–2018). (DOCX)
