# Supplementary figures and images for: Incidence and risk of venous thromboembolism according to primary treatment in women with ovarian cancer: A retrospective cohort study
Source: PLoS One. 2021 Apr 28;16(4):e0250723. doi: 10.1371/journal.pone.0250723 (PMC8081178; doi:10.1371/journal.pone.0250723)

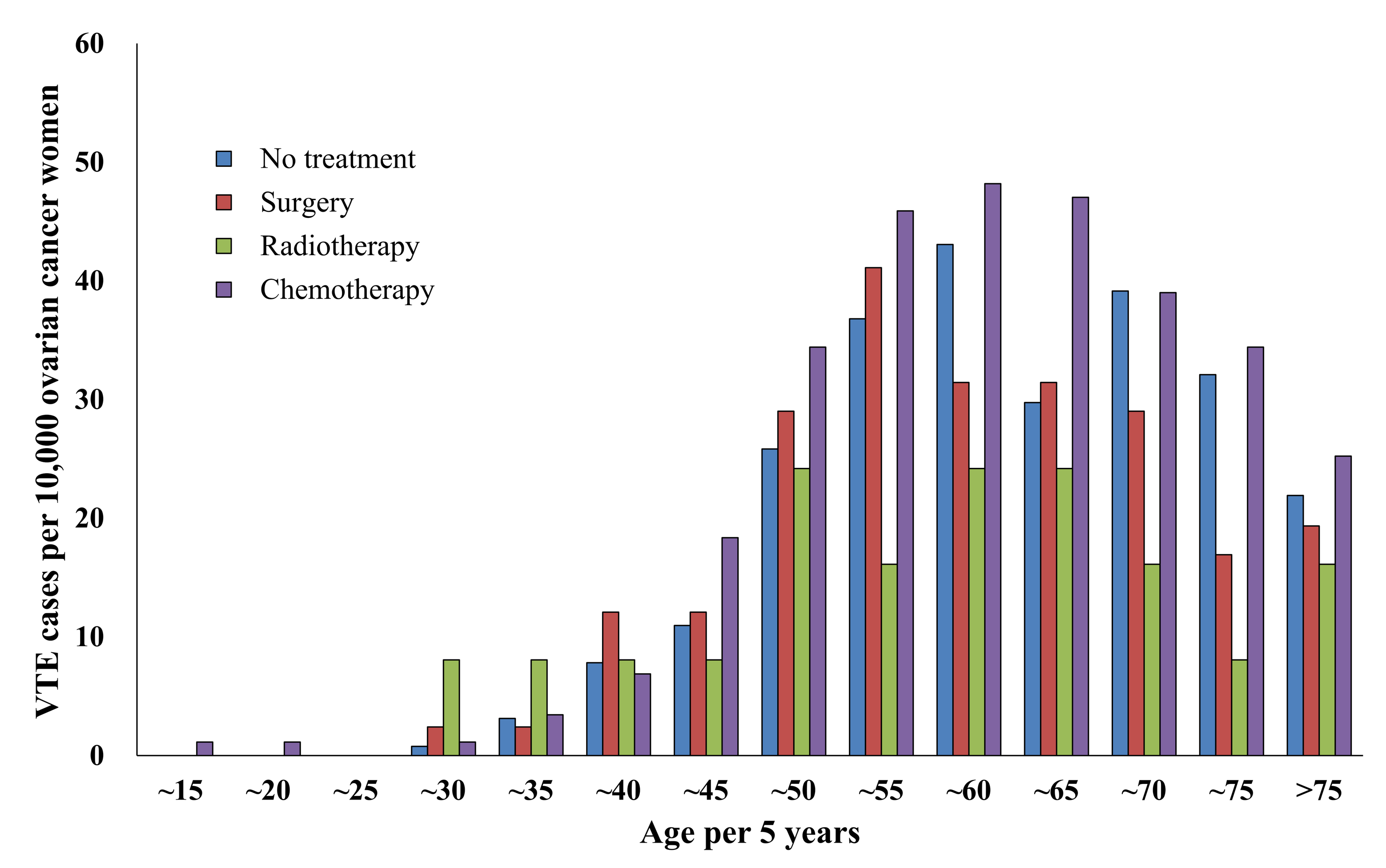

Supplement: S1 Fig — (TIF) [file pone.0250723.s001.tif]
