# Supplementary material for: Incidence and risk of venous thromboembolism according to primary treatment in women with ovarian cancer: A retrospective cohort study
Source: PLoS One. 2021 Apr 28;16(4):e0250723. doi: 10.1371/journal.pone.0250723 (PMC8081178; doi:10.1371/journal.pone.0250723)
Supplement: S1 Table — (DOCX) [file pone.0250723.s002.docx]

**S1 Table. Incidence of VTE according to pharmacologic thromboprophylaxis in women with ovarian cancer (HIRA claims data of 2009-2018).**

|  | VTE | | | | DVT | | | | PE | | | |
| --- | --- | --- | --- | --- | --- | --- | --- | --- | --- | --- | --- | --- |
|  | No | Yes | Total | P-value | No | Yes | Total | P-value | No | Yes | Total | P-value |
| Pharmacologic thromboprophylaxis |  |  |  | <0.001 |  |  |  | <0.001 |  |  |  | <0.001 |
| No | 15,236 (58.2) | 332 (47.3) | 15,568 (58) |  | 15,429 (58.1) | 139 (45) | 15,568 (58) |  | 15,354 (58.1) | 214 (49.8) | 15,568 (58) |  |
| Yes | 10,925 (41.8) | 370 (52.7) | 11,295 (42) |  | 11,125 (41.9) | 170 (55) | 11,295 (42) |  | 11,079 (41.9) | 216 (50.2) | 11,295 (42) |  |

DVT, deep vein thrombosis; PE, pulmonary embolism; VTE, venous thromboembolism.

All values ​​are expressed as number (%).
