# Supplementary material for: Incidence and risk of venous thromboembolism according to primary treatment in women with ovarian cancer: A retrospective cohort study
Source: PLoS One. 2021 Apr 28;16(4):e0250723. doi: 10.1371/journal.pone.0250723 (PMC8081178; doi:10.1371/journal.pone.0250723)
Supplement: S2 Table — (DOCX) [file pone.0250723.s003.docx]

**S2 Table. Methods for prophylaxis and treatment of VTE in women with ovarian cancer (HIRA claims data of 2009-2018).**

|  | Prophylaxis (n=11,295) | Treatments (n=702) |
| --- | --- | --- |
| LMWH | 777 (6.9) | 129 (18.4) |
| UFH | 5,557 (49.2) | 387 (55.1) |
| Warfarin | 744 (6.6) | 263 (37.5) |
| Aspirin | 5,762 (51.0) | 246 (35.0) |
| DOAC | 924 (8.2) | 414 (59.0) |
| Fondaparinux | 1 (0.0) | (0) |
| Thrombectomy |  | 2 (0.3) |
| Thromboplasty |  | (0) |
| Thrombolysis |  | 1 (0.1) |
| IVC filter |  | 37 (5.3) |

DOAC, direct oral anticoagulants; IVC, Inferior Vena Cava; LMWH, low molecular weight heparin; UFH, unfractionated heparin; VTE, venous thromboembolism

All values ​​are expressed as number (%).
